# Development and validation of a prognostic nomogram for adult patients with renal sarcoma: A retrospective study based on the SEER database

**DOI:** 10.3389/fpubh.2022.942608

**Published:** 2022-09-12

**Authors:** Yongkun Zhu, Weipu Mao, Guangyuan Zhang, Si Sun, Shuchun Tao, Tiancheng Jiang, Qingbo Wang, Yuan Meng, Jianping Wu, Ming Chen

**Affiliations:** ^1^Department of Urology, Affiliated Zhongda Hospital of Southeast University, Nanjing, China; ^2^Department of Medical College, Southeast University, Nanjing, China; ^3^Department of Chemotherapy, Affiliated the Second Hospital of Nanjing, Nanjing University of Chinese Medicine, Nanjing, China; ^4^Department of Urology, Nanjing Lishui People's Hospital, Zhongda Hospital Lishui Branch of Southeast University, Nanjing, China

**Keywords:** adult patients, renal sarcoma, nomogram, SEER, prognosis

## Abstract

**Background:**

Renal sarcoma (RS) is rarely seen in clinical practice. The purpose of this study was to develop a prognostic nomogram model, which could predict the probability of overall survival (OS) and cancer-specific survival (CSS) in adult patients with RS.

**Methods:**

Patients diagnosed with RS were recruited from the SEER database between 2004 and 2015, and randomized to two cohorts: the training cohort and the validation cohort. Uni- and multivariate Cox regression analyses in the training cohort were used to screen independent prognostic factors for OS and CSS. Prognostic nomograms for OS and CSS were created separately for adult RS patients based on independent risk factors. The area under the receiver operating characteristic (ROC) curves, calibration curves, and decision curve analysis (DCA) were used to validate the nomograms.

**Results:**

A total of 232 eligible patients were recruited, including 162 in the training cohort and 70 in the validation cohort. Sex, histological type, SEER stage, and surgery were independent prognostic factors for OS, while histological type, SEER stage, surgery, chemotherapy were independent prognostic factors for CSS. Based on the above independent prognostic factors, prognostic nomograms for OS and CSS were created respectively. In the training cohort, the AUCs of the nomograms for OS and CSS were 0.742 and 0.733, respectively. In the validation cohort, the AUCs of the nomograms for OS and CSS were 0.837 and 0.758, respectively. The calibration curves of the nomograms showed high consistencies between the predicted and actual survival rates. Finally, the DCA demonstrated that the nomograms in the wide high-risk threshold had a higher net benefit than the SEER stage.

**Conclusion:**

A prognostic nomogram for renal sarcoma was created and validated for reliability and usefulness in our study, which assisted urologists in accurately assessing the prognosis of adult RS patients.

## Introduction

Sarcomas are a heterogeneous group of tumors arising in the embryonic mesoderm, accounting for approximately 1% of all malignant tumors, of which <5% occur in the urogenital tract (1). Primary renal sarcoma (RS) accounts for around 24.6% of all genitourinary sarcomas and <1% of all primary kidney tumors (1, 2). Renal sarcoma is not only very rare but also leads to a poor prognosis: the overall 1-, 3-, and 5-year survival rate was 86.3, 40.7, and 14.5%, respectively, and the median survival was 28 months (3). According to previous reviews and case reports, renal sarcoma could be classified into the following pathological types: liposarcoma (4), leiomyosarcoma (5), carcinosarcoma (6), rhabdomyosarcoma (7), clear cell sarcoma (8), fibrosarcoma (9) and others, and different pathological types predict distinct prognosis.

RS is currently poorly studied as it is such a rare malignancy. As a result, an accurate prognostic model for RS is essential for both urologists and patients. In fact, the SEER stage grading system was employed by urologists to measure the progression of RS, which includes localized, regional, distant, and unstaged (10, 11). However, other factors including sex, age, year of diagnosis, race, marital status, radiation, chemotherapy, surgery, etc. may also have an impact on prognosis due to individual variances. In recent years, nomograms have been increasingly employed in clinical practice for cancer prognosis. It has been regarded as a useful statistical prediction tool for benefiting both clinicians and patients (12, 13). So far, there is no report on the application of nomograms in predicting the prognosis of renal sarcoma in adults. In the present study, based on data from the SEER database between 2004 and 2015, nomograms were set up to predict survival outcomes for adult patients with RS and their reliability was also validated.

## Materials and methods

### Data sources

Data were extracted from the Surveillance Epidemiology and End Results (SEER) database (https://seer.cancer.gov/), which is supported by the Surveillance Research Program (SRP) in NCI's Division of Cancer Control and Population Sciences (DCCPS). SEER statistics are collected on a national scale, with information from 18 states that represent all regions of the country covering 28% of the US population, including sociodemographic factors, geographic variables, clinical factors, cancer-specific factors, pathologic variables, treatment factors, and outcomes (14). The SEER database is openly accessed, and all authors have obtained permission. SEER^*^Stat software [Version 8.3.9.2 - August 20, 2021, SEER^*^Stat Software (cancer.gov)] was used to extract the data.

### Patients

**Figure 1:**
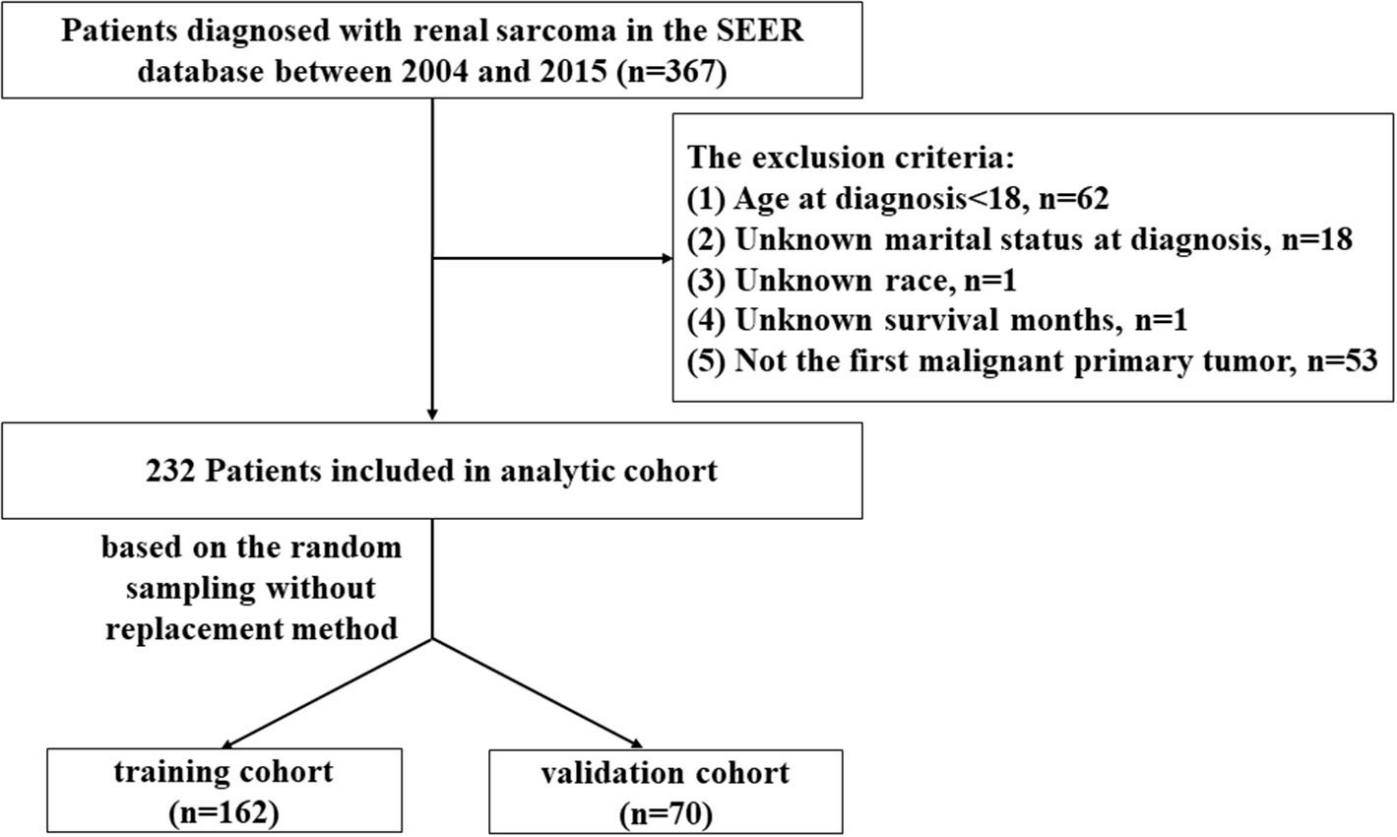
The study flow chart of the selection process.

A total of 367 patients diagnosed with RS between 2004 and 2015 were established according to the International Classification of Disease for Oncology, Third Edition [ICD-O-3] site codes, including liposarcoma (8850/3, 8851/3, 8852/3, 8853/3, 8858/3, 8860/3), leiomyosarcoma (8890/3, 8891/3, 8896/3), carcinosarcoma (8980/3), rhabdomyosarcoma (8900/3, 8901/3, 8910/3), clear cell sarcoma (8964/3), fibrosarcoma (8810/3), sarcoma, NOS (8800/3). The exclusion criteria are based on the following principles: (1) age at diagnosis is below 18 years old, *n* = 62; (2) unknown marital status at diagnosis, *n* = 18; (3) unknown Race, *n* = 1; (4) unknown Survival months, *n* = 1; (5) not the first malignant primary tumor, *n* = 53. Finally, 232 eligible patients were included in the analytic cohort. The flow chart of the selection process was presented in Figure 1.

### Variables and endpoints

The following variables were filtered from the SEER database: age, year of diagnosis, sex, marital status, race, grade, histological type, SEER stage, surgery, radiotherapy, chemotherapy. To facilitate the next step of data analysis, the categorical variables were coded directly, and for continuous variables, they were first converted to categorical variables before coding. Some of the variables are explained below:

1. Regarding age, patients were divided into two categories: older than 60 years and ≤ 60.
2. Regarding year of diagnosis, it was divided into two phases: 2004–2009, 2010–2015.
3. Regarding grade, it was defined as follows: well-differentiated (Grade I); moderately differentiated (Grade II); poorly differentiated (Grade III); undifferentiated (Grade IV); and unknown grade.
4. Regarding the stage of SEER, patients were classified into four subgroups according to the progression of the sarcoma, including localized, regional, distant, and unstaged.

The death and RS-specific death were regarded as observed endpoints. OS refers to the period between the start of the study and death from any cause, and survivors are censored as of the last follow-up. CSS refers to the period between the commencement of the study and the death due to RS, with deaths due to other causes or survivors omitted.

### Statistical methods

Categorical data were described as numbers (*n*) and percentages (%), and chi-square tests were used to assess differences in categorical variables. The sample was divided into a training cohort and a validation cohort (in a ratio of 7:3) using a no-replacement random sampling method. The training cohort was used to create nomograms and filter factors for nomograms, while the validation cohort was used to validate the results of the training cohort. Univariate Cox regression was used to identify factors associated with OS and CSS, and multivariate Cox regression to identify associated independent risk factors. Variables with *P* values <0.05 in univariate Cox regression analysis were included in multivariate Cox regression analysis, and associated hazard ratios (HR) and 95% confidence intervals (CI) were calculated. Based on the results of multivariate Cox regression analysis, independent risk factors were used to create prognostic nomograms to predict the probability of OS and CSS at 3 and 5 years. In addition, receiver operating characteristic (ROC) curves, decision curve analysis (DCA), and calibration curves were used to assess the predictive performance of the nomogram and SEER stage.

A vertical line was drawn on the scale for each variable for a given adult RS patient, and the intersection with the “dot” line represented the score for that variable. The total score is calculated by adding up the scores for each variable. Matching scores were found on the “total score” line and projected onto the OS and CSS lines below, resulting in 3- and 5-OS and CSS probabilities for that individual.

In ROC curve analysis, the area under the curve (AUC) is defined as the area enclosed by the ROC curve and the coordinate axes. The value of the AUC usually ranges between 0.5 and 1, and the diagnostic value of the nomogram is represented by the AUC. In the calibration curve analysis, a bootstrap method with 1,000 resamples was used for testing.

SPSS 26.0 (IBM Corp. Released 2019. IBM SPSS Statistics for Windows, Version 26.0. Armonk, NY: IBM Corp.) was applied to conduct statistical analysis for univariate and multivariate Cox regression. The nomograms were developed and validated by exerting the rms, hmisc, lattice, survival, formula, ggplot2, pROC, timeROC, and rmda packages in R version 4.1.2 (http://www.r-project.org/). *P* <0.05 (two-sided) was considered statistically significant.

## Results

### Baseline demographic and clinical characteristics

A total of 232 eligible patients diagnosed with RS between 2004 and 2015 were included in our study, which were divided into two cohorts randomly: the training cohort (162, 70.0%) and the validation cohort (70, 30.0%). The number of RS patients aged over or equal to 60 and under 60 was similar in the total cohort. Similarly, the number of patients with the year of diagnosis in 2004–2009 and in 2010–2015 was approximately equal. Most RS patients were female (54.7%), married (57.3%), and white (81.1%). Grade IV accounts for the largest proportion of known grades. Of the other general type, the majority were leiomyosarcoma (40.9%) and localized (34.1%). Most RS adult patients received radiotherapy (85.3%) and chemotherapy (76.7%), but only a few had undergone surgery (22.4%). Specific baseline demographic and clinical characteristics information are represented in Table 1.

**Table 1:** Baseline demographic and clinical characteristics with adult renal sarcoma patients in our study.

**Characteristic**	**Total no. (%)**	**The trainingcohort**	**The validation cohort**	***P value***
		**No. (%)**	**No. (%)**	
Total	232 (100)	162 (70.0)	70 (30.0)	
Age, years				0.219
≤ 60	120 (51.7)	79 (48.8)	41 (58.6)	
>60	112(48.3)	83 (51.2)	29 (41.4)	
Year of diagnosis				0.627
2004–2009	115 (49.6)	82 (50.6)	33 (47.1)	
2010–2015	117 (50.4)	80 (49.4)	37 (52.9)	
Sex				0.814
Male	105 (45.3)	72 (44.4)	33 (47.1)	
Female	127 (54.7)	90 (55.6)	37 (52.9)	
Marital status				0.330
Married	133 (57.3)	89 (54.9)	44 (62.9)	
Unmarried	99 (42.7)	73 (45.1)	26 (37.1)	
Race				0.606
White	188 (81.1)	134 (82.7)	54 (77.1)	
Black	27 (11.6)	17 (10.5)	10 (14.3)	
Others	17 (7.3)	11 (6.8)	6 (8.6)	
Grade				0.878
Grade I	22 (9.5)	14 (8.5)	8 (11.4)	
Grade II	22 (9.5)	16 (9.9)	6 (8.6)	
Grade III	39 (16.8)	28 (17.3)	11 (15.7)	
Grade IV	67 (28.9)	49 (30.2)	18 (25.7)	
Unknown	82 (35.3)	55 (34.0)	27 (38.6)	
Histological type				0.308
Liposarcoma	69 (29.7)	52 (32.1)	17 (24.3)	
Leiomyosarcoma	95 (40.9)	60 (37.0)	35 (50.0)	
Carcinosarcoma	10 (4.3)	6 (3.7)	4 (5.7)	
Rhabdomyosarcoma	4 (1.7)	3 (1.9)	1 (1.4)	
Clear cell sarcoma	19 (8.3)	12 (7.4)	7 (10.0)	
Fibrosarcoma	2 (0.9)	2 (1.2)	0	
Sarcoma, NOS	33 (14.2)	27 (16.7)	6 (8.6)	
SEER stage				0.178
Localized	79 (34.1)	56 (34.6)	23 (32.9)	
Regional	70 (30.2)	43 (26.5)	27 (38.6)	
Distant	73 (31.5)	54 (33.3)	19 (27.1)	
Unstaged	10 (4.2)	9 (5.6)	1 (1.4)	
Surgery				0.274
Yes	52 (22.4)	40 (24.7)	12 (17.1)	
No/Unknown	180 (77.6)	122 (75.3)	58 (82.9)	
Radiotherapy				1.000
Yes	198 (85.3)	138 (85.2)	60 (85.7)	
No/Unknown	34 (14.7)	24 (14.8)	10 (14.3)	
Chemotherapy				0.788
Yes	178 (76.7)	123 (75.9)	55 (78.6)	
No/Unknown	54 (23.3)	39 (24.1)	15 (21.4)	

SEER, Surveillance, Epidemiology, and End Results. Percentages may not total 100 because of rounding.

### Univariate and multivariate analysis of OS and CSS

The univariate and multivariate Cox regression analysis of OS and CSS rates in the training cohort was carried out for screening independent prognostic variables. Age, year of diagnosis, sex, marital status, race, grade, histological type, SEER stage, surgery, radiotherapy, and chemotherapy were included in our analysis. By univariate regression analysis, it was shown that all variables mentioned above might be substantially linked with OS and CSS. Meanwhile, it was also shown that sex, histological type, SEER stage, and surgery were independent predictive variables for OS by multivariate analysis, while histological type, SEER stage, surgery, and chemotherapy were independent prognostic variables for CSS. Confidence intervals (CI) and corresponding *p*-values for specific variables in the univariate and multivariate analyses of OS and CSS were summarized in Tables 2, 3, respectively.

**Table 2:** Univariate and multivariate analysis of overall survival (OS) rates in the training cohort.

**Characteristic**	**Univariate analysis**		**Multivariate analysis**	
	**Hazard ratio (95% CI)**	***P* value**	**Hazard ratio (95% CI)**	***P* value**
Age, years				
≤ 60	Reference		Reference	
>60	1.469 (1.000–2.159)	0.050	-	0.098
Year of diagnosis				
2004–2009	Reference			
2010–2015	0.652 (0.418–1.015)	0.058		
Sex				
Male	Reference		Reference	
Female	0.601 (0.409–0.881)	0.009	0.498 (0.328–0.756)	0.001
Marital status				
Married	Reference		Reference	
Unmarried	0.869 (0.592–1.277)	0.475	-	0.745
Race				
White	Reference		Reference	
Black	0.453 (0.219–0.935)	0.032	-	0.038
Others	1.217 (0.561–2.641)	0.619	-	0.868
Grade				
Grade I	Reference		Reference	
Grade II	1.489 (0.355–6.240)	0.586	-	0.091
Grade III	3.301 (0.960–11.349)	0.058	-	0.752
Grade IV	5.251 (1.619–17.025)	0.006	-	0.205
Unknown	5.779 (1.791–18.647)	0.003	-	0.498
Histological type				
Liposarcoma	Reference		Reference	
Leiomyosarcoma	1.364 (0.839–2.219)	0.210	1.406 (0.854–2.315)	0.181
Carcinosarcoma	7.253 (2.936–17.919)	<0.001	6.996 (2.703-18.107)	<0.001
Rhabdomyosarcoma	2.590 (0.783–8.562)	0.119	3.797 (1.127–12.789)	0.031
Clear cell sarcoma	1.063 (0.480–2.353)	0.880	0.542 (0.232–1.266)	0.157
Fibrosarcoma	0.701 (0.095–5.170)	0.728	0.374 (0.050–2.809)	0.339
Sarcoma, NOS	1.910 (1.061–3.437)	0.031	1.563 (0.843–2.898)	0.157
SEER stage				
Localized	Reference		Reference	
Regional	2.769 (1.599–4.794)	<0.001	3.623 (2.047–6.410)	<0.001
Distant	4.793 (2.861–8.029)	<0.001	4.317 (2.487–7.494)	<0.001
Unstaged	2.444 (0.924-6.462)	0.072	1.936 (0.645-5.805)	0.239
Surgery				
No/Unknown	Reference		Reference	
Yes	0.478 (0.313–0.728)	0.001	0.515 (0.313–0.847)	0.009
Radiotherapy				
Yes	Reference		Reference	
No/Unknown	0.875 (0.521–1.471)	0.615	-	0.771
Chemotherapy				
Yes	Reference		Reference	
No/Unknown	0.679 (0.444–1.038)	0.074	-	0.348

CSS, Cancer-specific survival; SEER, Surveillance, Epidemiology, and End Results; HR, hazard ratio; CI, confidence interval.

**Table 3:** Univariate and multivariate analysis of cancer-specific survival (CSS) rates in the training cohort.

**Characteristic**	**Univariate analysis**		**Multivariate analysis**	
	**Hazard ratio (95% CI)**	***P* value**	**Hazard ratio (95% CI)**	***P* value**
Age, years				
≤ 60	Reference		Reference	
>60	2.101 (1.144–3.859)	0.017	-	0.083
Year of diagnosis				
2004–2009	Reference			
2010–2015	0.761 (0.557–1.040)	0.087		
Sex				
Male	Reference		Reference	
Female	0.838 (0.466–1.507)	0.556	-	0.767
Marital status				
Married	Reference		Reference	
Unmarried	0.714 (0.394–1.294)	0.267	-	0.256
Race				
White	Reference		Reference	
Black	0.538 (0.192–1.508)	0.238	-	0.433
Others	0.749 (0.180–3.117)	0.691	-	0.652
Grade				
Grade I	-		-	
Grade II	Reference		Reference	
Grade III	2.378 (0.493–11.461)	0.280	-	0.919
Grade IV	4.481 (1.044–19.228)	0.044	-	0.153
Unknown	3.624 (0.836–15.713)	0.085	-	0.698
Histological type				
Liposarcoma	Reference		Reference	
Leiomyosarcoma	2.088 (0.802–5.437)	0.132	2.225 (0.839–5.901)	0.108
Carcinosarcoma	24.382 (7.227–82.262)	<0.001	23.815 (6.516–87.039)	<0.001
Rhabdomyosarcoma	3.799 (0.456–31.661)	0.217	9.022 (0.995–81.826)	0.051
Clear cell sarcoma	3.740 (1.198–11.676)	0.023	2.686 (0.825–8.740)	0.101
Fibrosarcoma	3.026 (0.363–25.224)	0.306	4.303 (0.446–41.551)	0.207
Sarcoma, NOS	5.748 (2.165–15.262)	<0.001	4.816 (1.712–13.547)	<0.001
SEER stage				
Localized	Reference		Reference	
Regional	3.106 (1.214–7.948)	0.018	3.926 (1.492–10.328)	0.006
Distant	7.031 (2.988–16.547)	<0.001	5.867 (2.301–14.962)	<0.001
Unstaged	4.656 (1.201–18.049)	0.026	1.800 (0.379–8.557)	0.460
Surgery				
No/Unknown	Reference		Reference	
Yes	0.350 (0.191–0.639)	0.001	0.352 (0.168–0.739)	0.006
Radiotherapy				
Yes	Reference		Reference	
No/Unknown	0.950 (0.425–2.127)	0.901	-	0.518
Chemotherapy				
Yes	Reference		Reference	
No/Unknown	0.889 (0.450–1.756)	0.735	2.315 (1.065–5.033)	0.034

CSS, Cancer-specific survival; SEER, Surveillance, Epidemiology, and End Results; HR, hazard ratio; CI, confidence interval.

### Nomogram development and validation

**Figure 2:**
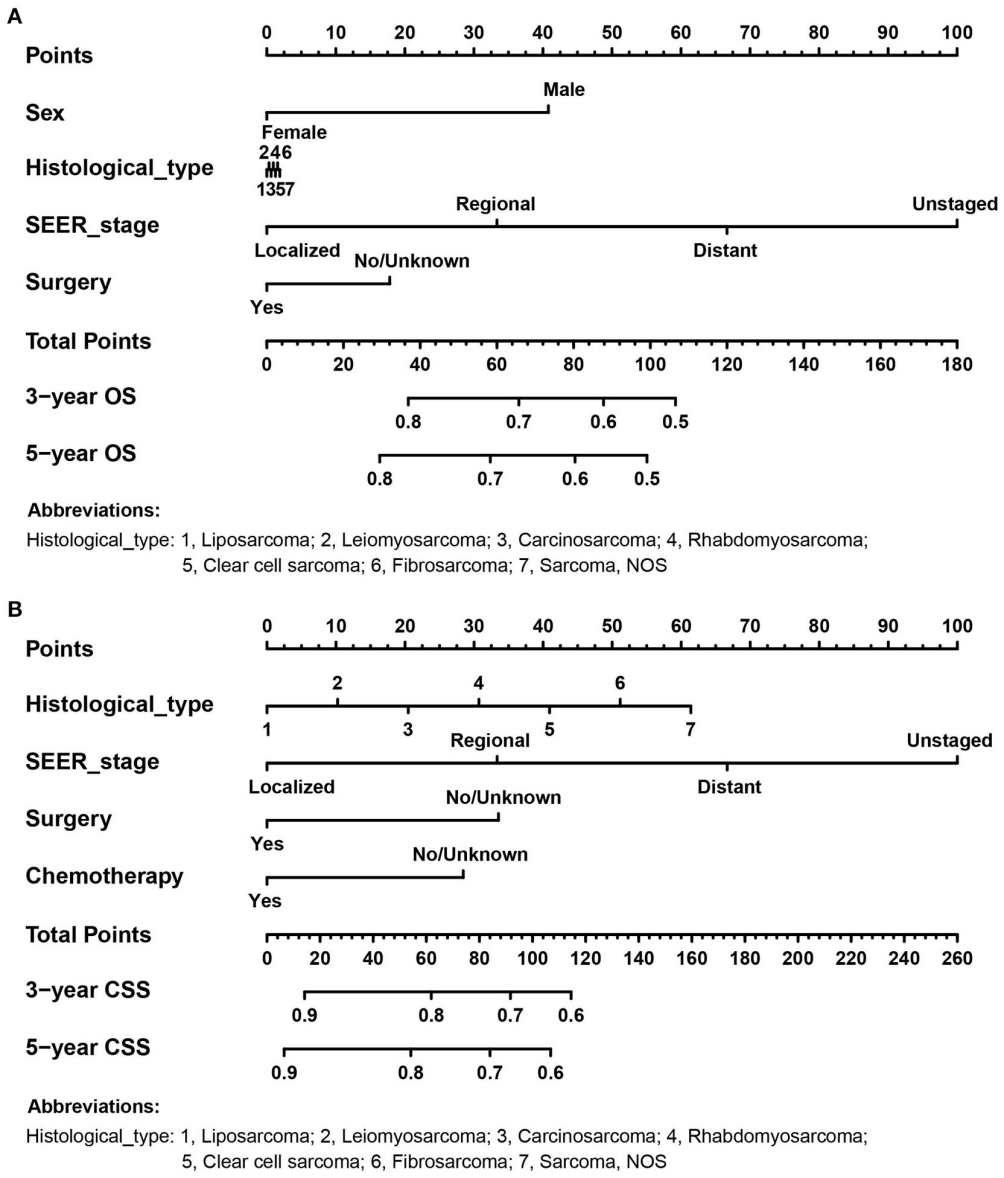
The prognostic nomograms for predicting 3- and 5- OS and CSS probabilities of adult RS patients in the training cohort. **(A)** OS nomogram; **(B)** CSS nomogram.

According to the independent prognostic variables of OS and CSS, the nomograms were established, respectively (Figure 2). In the OS nomogram, the SEER stage contributed the most to survival outcome, while the histological type contributed the least. In the CSS nomogram, the SEER stage was the most significant predictor of survival, followed by histological type.

**Figure 3:**
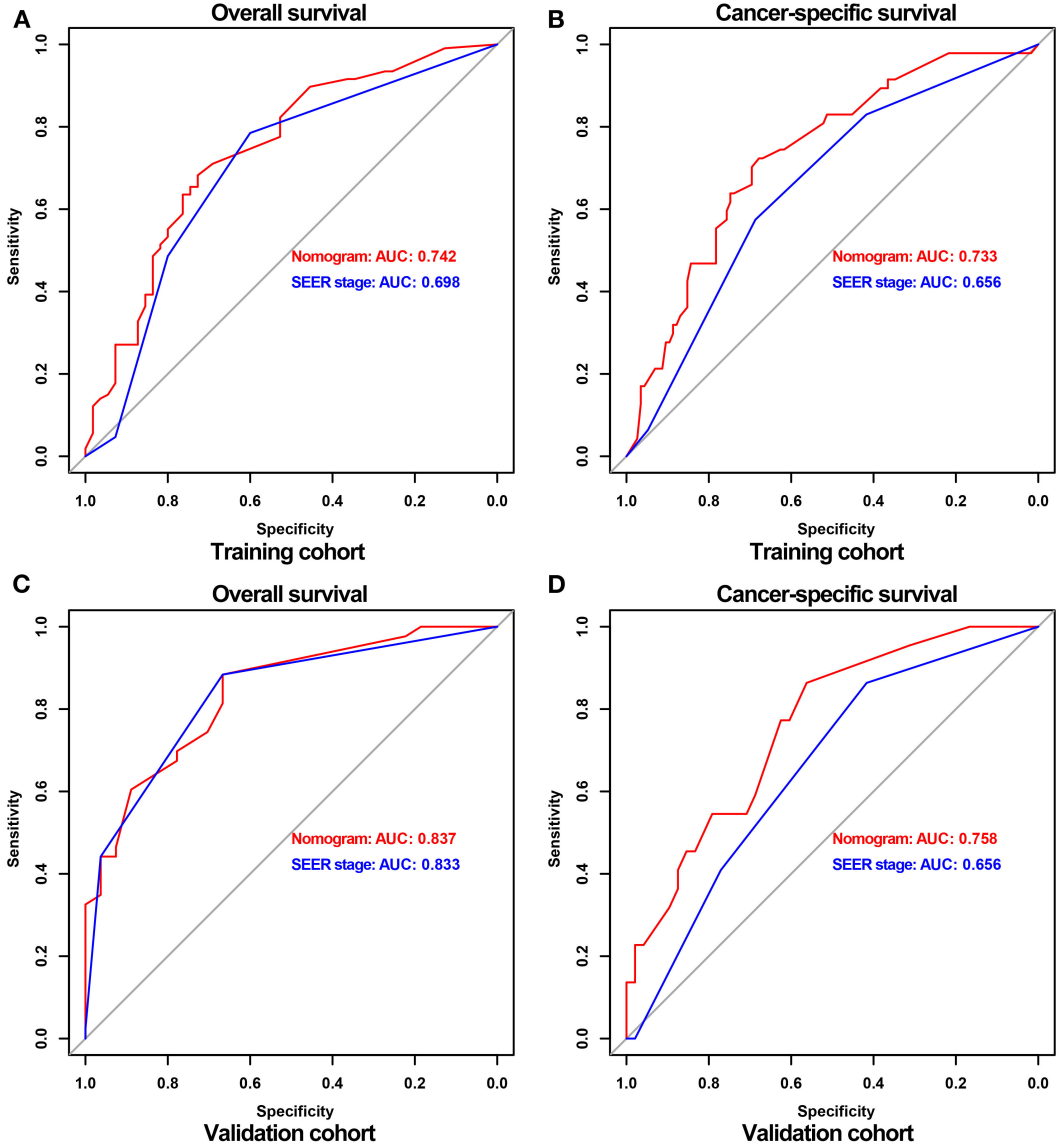
ROC curves of nomograms and the SEER stage for predicting OS and CSS probabilities in the training and validation cohort. ROC for OS **(A)** and CSS **(B)** in the training cohort, respectively; ROC for OS **(C)** and CSS **(D)** in the validation cohort, respectively.

As shown in Figure 3, the ROC curves were drawn, and the AUC of the OS nomogram was significantly greater than that of the SEER stage in the training cohort (nomogram 0.742, SEER stage 0.698), while in the validation cohort the AUC of the OS nomogram was similar to SEER stage (nomogram 0.837, SEER 0.833). However, the AUCs of the nomograms for CSS were considerably higher than those of the SEER stage both in the training cohort (nomogram 0.733, SEER stage 0.656) and validation cohort (nomogram 0.758, SEER stage 0.656). By comprising of the above ROC curves, it was demonstrated that the nomogram had more diagnostic value than the SEER stage to discriminate the survival probability of adult RS patients.

**Figure 4:**
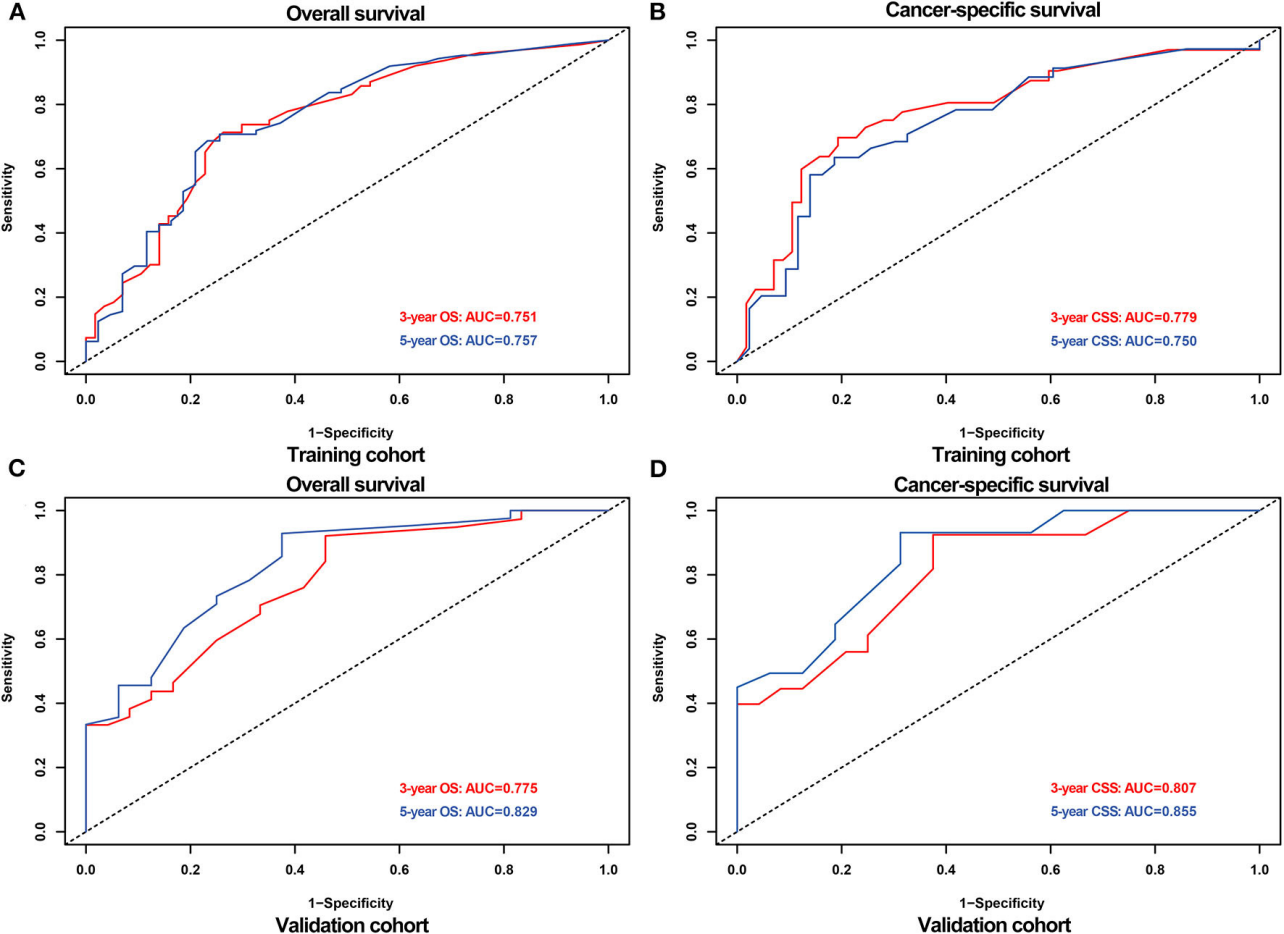
ROC curves for predicting 3-,5- OS and CSS probabilities in the training and validation cohort. ROC for 3-,5- OS **(A)** and CSS **(B)** in the training cohort; ROC for 3-,5- OS **(C)** and CSS **(D)** in the validation cohort.

The AUCs for 3- and 5-OS were 0.751 and 0.757, respectively, and 0.779 and 0.750 for 3- and 5-CSS, respectively, in the training cohort. The validation cohort AUCs for 3- and 5-OS were 0.775 and 0.829, respectively, and 0.807 and 0.855, respectively, for 3- and 5-CSS. As shown in Figure 4, the nomograms accurately predict the probability of 3- and 5- OS and CSS for adult RS patients.

**Figure 5:**
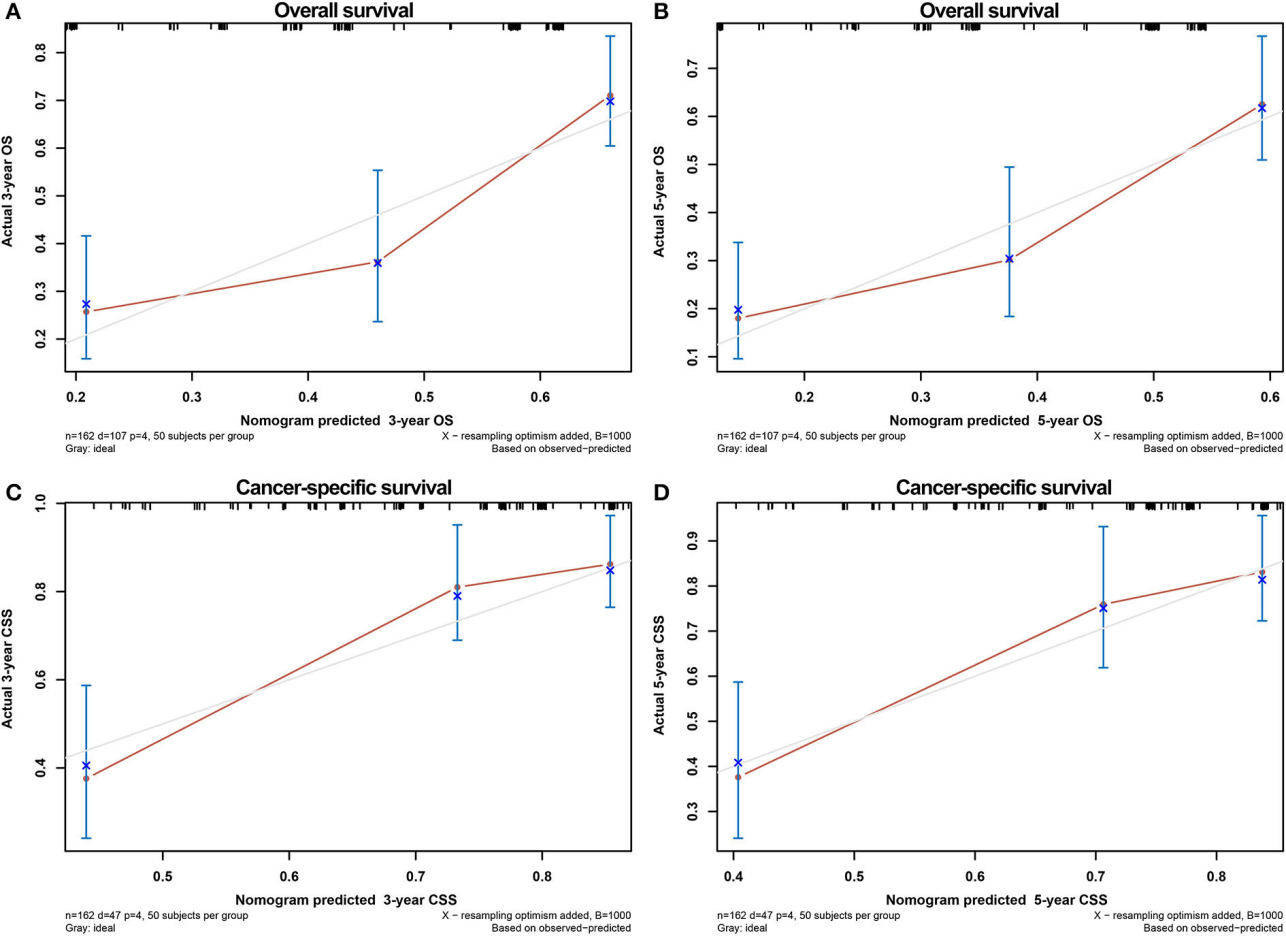
Calibration curves for verifying the consistency between predicted 3-,5- OS and CSS and actual 3-,5- OS and CSS in the training cohort. 3- OS **(A)** and 5- OS **(B)** calibration curves; 3- CSS **(C)** and 5- CSS **(D)** calibration curves.

**Figure 6:**
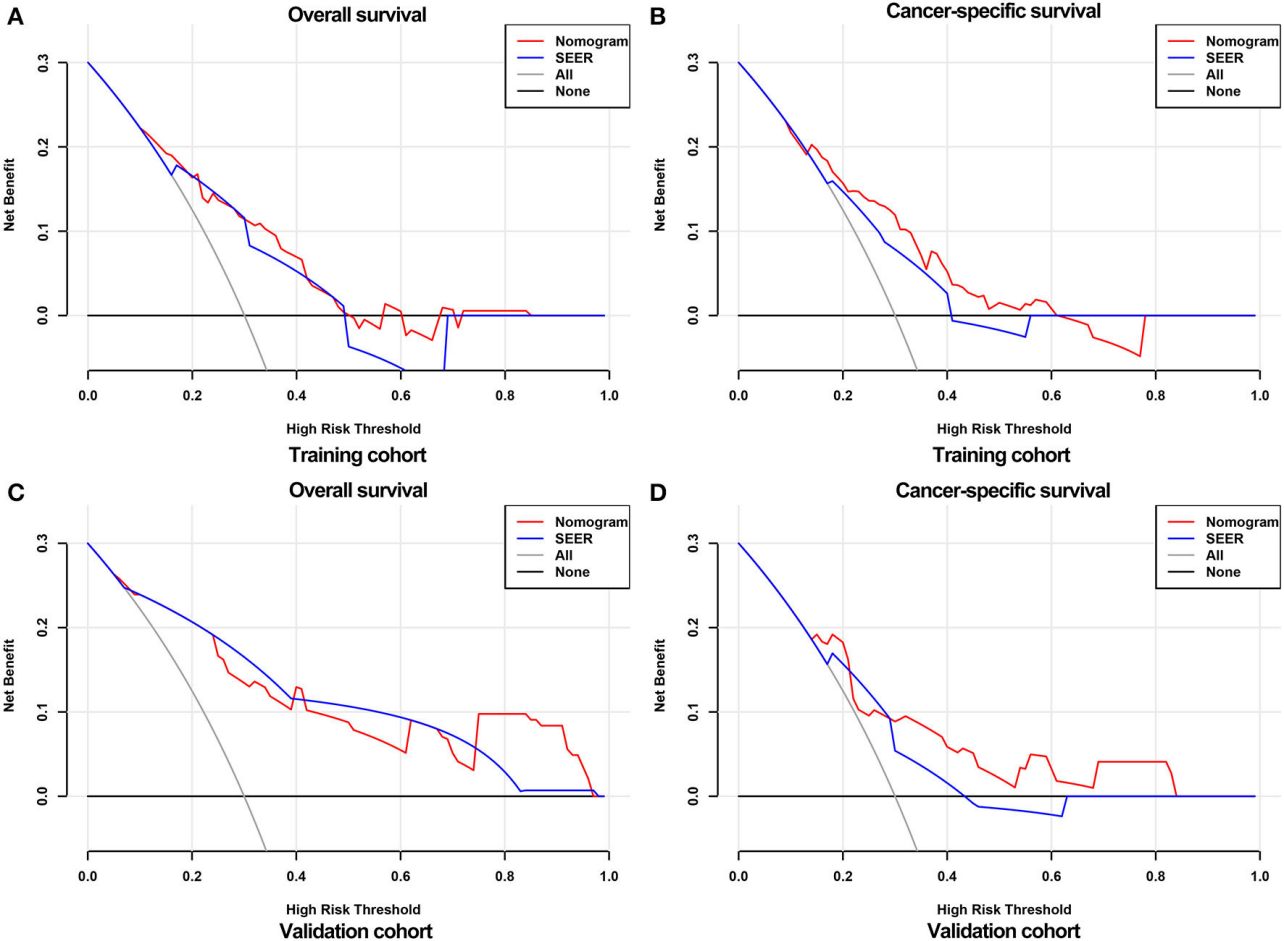
DCA curves for validating the clinical utility of the nomograms. DCA curves for OS **(A)** and CSS **(B)** in the training cohort. DCA curves for OS **(C)** and CSS **(D)** in the validation cohort.

The calibration curves of the nomograms showed high consistencies between the predicted and actual survival rates both in the training and validation cohorts, illustrated in Figure 5 and Supplementary Figure 1. The gray line in the calibration curves represents the ideal reference line, where the predicted survival probability matches the actual survival probability. The presentation of the nomograms was represented by red dots. The DCA demonstrated that the nomograms in the wide high-risk threshold had a higher net benefit than the SEER stage (Figure 6), which validated the superiority of the nomogram utility over the SEER stage in clinical practice.

## Discussion

As mentioned above, adult renal sarcomas are an extremely rare group of tumors, accounting for only 0.8% of primary renal tumors (3). The SEER stage grading system was used by urologists to evaluate the progression of renal sarcomas. Sarcomas are classified into different grades based on the location and the extent to which it invades organs, blood vessels, and lymph nodes, including localized, regional, distant, and unstaged. However, due to the influence of individual differences, such as sex, age, race, marital status, radiation, chemotherapy, surgery, etc., it is not comprehensive enough to use the extent of tumor invasion alone to evaluate the prognosis for adult RS patients.

The nomogram is a graphical representation of a clinical prediction model that calculates a total score based on the values of individual predictor variables, and then predicts the risk of an event or the probability of survival based on the total score (15, 16). It is a novel prediction model that is gradually sought after by clinicians. In recent years, predictions for the prognosis of various urinary cancer with nomograms have been reported more and more. For instance, Wu et al. employed a genomic-clinicopathologic nomogram to predict preoperative lymph node metastasis in bladder cancer (17); A nomogram was conducted by Mao et al. to predict prognosis in patients with lung metastatic renal cell carcinoma (18). Zhang et al. established a radiomics nomogram to predict bone metastasis in newly diagnosed prostate cancer patients (19). The nomogram and Aggtrmmns scoring system were utilized by Zhou et al. for predicting overall survival and cancer-specific survival of kidney cancer patients (20).

As it is known that compared with the SEER stage, nomogram has the following advantages: (1) By combining various independent risk factors according to the patient's condition, it allows for a more intuitive assessment and individualization of the patient's prognosis (21). (2) It quantifies the possibility of OS and CSS in patients, permitting a more precise prognostic evaluation (22). Therefore, for the first time, the prognostic nomograms were developed for adult RS patients to obtain personalized and accurate prognostic predictions in this study.

We extracted data from the SEER database for adult RS patients and used COX univariate and subsequent multivariate regression analysis to conclude that histological type, SEER stage, surgery were independent risk factors for OS and CSS. Based on the multivariate regression analysis, the OS and CSS nomograms were constructed, respectively. Subsequently, we validated the nomograms. The area under the ROC curves for 3-,5- OS were 0.775 and 0.829, respectively, and 0.807 and 0.855 for 3-, 5- CSS, respectively, which depicted that the nomograms accurately predict the probability of 3- and 5- OS and CSS for adult RS patients. The calibration curves showed high consistencies between the predicted and actual survival rates.

From the nomograms, it was suggested that RS patients without surgery, with distant SEER stage grade, and histological type of carcinosarcoma had the poorest prognosis. According to the Kaplan-Meier overall and disease-specific survival analysis of patients with RS established by Nazemi et al. (1), liposarcoma had the greatest prognosis, followed by leiomyosarcoma and clear cell sarcoma, while carcinosarcoma had the worst prognosis. Some studies have shown that carcinosarcoma had the worst prognosis, which was consistent with our analysis. In addition, in our study, univariate Cox regression analysis found that Sarcoma, NOC patients had poorer OS compared to liposarcoma patients (HR = 1.910, 95% CI 1.061–3.437, *p* = 0.031). However, after multivariate Cox regression analysis, there was no difference between the two groups, which could be explained by the inclusion of other confounding variables, which led to biased results.

In addition, our study demonstrated that surgical treatment for adult RS patients may effectively reduce the risk of death. This is in line with the findings of Moreira et al. (14) and Öztürk (23). Moreover, it is also found that chemotherapy may also improve the prognosis of adult RS patients. Chemotherapy has now been applied clinically to treat advanced or recurrent renal sarcoma, although not standardized (24), and the latest research of Yakirevich et al. suggested that comprehensive genomic analysis of adult RS patients may provide new opportunities for targeted therapy (25).

To our surprise, our data suggested radiotherapy was not an independent prognostic factor for the adult patient with renal sarcoma, which was in accordance with the findings of Li et al. (26). However, Gamboa et al. reported that preoperative radiotherapy may improve the prognosis by making some tumors easier to resect (27). Thus, the prognostic impact of radiotherapy on patients with renal sarcomas should be further explored. The clinical outcome of primary adult renal sarcoma is extremely poor and the optimal treatment remains to be debated. Further studies are needed to verify whether it is surgery or combination therapy that works best. Furthermore, our data also suggested that female patients had a better prognosis than male patients, which could be attributed to differences in female anatomy or hormone levels.

We appraised the prognosis of adult RS patients with nomograms for the first time, which adds a new dimension to our research. Simultaneously, using the SEER database excluded the influencing factors of single-center. Even so, there are still a few flaws in our study: (1) Because of the rarity of renal sarcoma, limited sample size is inevitable and therefore our findings may not be representative; (2) As our study is retrospective, there is a lack of multicenter data for external validation. (3) Due to the lack of data in the SEER database, genetic factors, laboratory findings, and medication history were not included in our study.

## Conclusions

In conclusion, a prognostic nomogram was created to predict overall survival (OS) and cancer-specific survival (CSS) for adult patients with RS, and their reliability and usefulness were also validated in our study. We anticipate that our study will facilitate urologists in accurately assessing the prognosis of adult RS patients and provide support for further clinical trials.

## Data availability statement

The datasets presented in this study can be found in online repositories. The names of the repository/repositories and accession number(s) can be found in the article/Supplementary material.

## Author contributions

YZ, MC, JW, YM, and QW: study designing. MC, JW, YM, and QW: administrative support. YZ, WM, GZ, SS, ST, and TJ: data collection. YZ, WM, GZ, and SS: statistical analysis and graphs production. YZ, WM, and GZ: draft writing. YZ, WM, GZ, SS, ST, TJ, QW, YM, JW, and MC: final revision. All authors contributed to the article and approved the submitted version.

## Funding

This study was funded by Natural Science Foundation of China (82170703 to GZ and 82100732), Natural Science Foundation of Jiangsu Province (BK20200360), and Excellent Youth Development Fund of Zhongda Hospital, SEU (2021ZDYYYQPY04).

## Conflict of interest

The authors declare that the research was conducted in the absence of any commercial or financial relationships that could be construed as a potential conflict of interest.

## Publisher's note

All claims expressed in this article are solely those of the authors and do not necessarily represent those of their affiliated organizations, or those of the publisher, the editors and the reviewers. Any product that may be evaluated in this article, or claim that may be made by its manufacturer, is not guaranteed or endorsed by the publisher.

## Supplementary material

Supplementary Figure 1 Calibration curves for verifying the consistency between predicted 3-,5- OS and CSS and actual 3-,5- OS and CSS in the validation cohort. 3- OS **(A)** and 5- OS **(B)** calibration curves; 3- CSS **(C)** and 5- CSS **(D)** calibration curves.

## Abbreviations

RS: Renal sarcoma
SEER, Surveillance, Epidemiology: and End Results
OS: Overall survival
CSS: Cancer-specific survival
DCA: Decision curve analysis
ICD-O: The International Classification of Diseases for Oncology
ROC: Receiver operating characteristic
AUC: Area under the curve
HR: Hazard ratios
CI: Confidence intervals.
