# Supplementary figures and images for: Development and validation of a prognostic nomogram for adult patients with renal sarcoma: A retrospective study based on the SEER database
Source: Front Public Health. 2022 Sep 12;10:942608. doi: 10.3389/fpubh.2022.942608 (PMC9524186; doi:10.3389/fpubh.2022.942608)

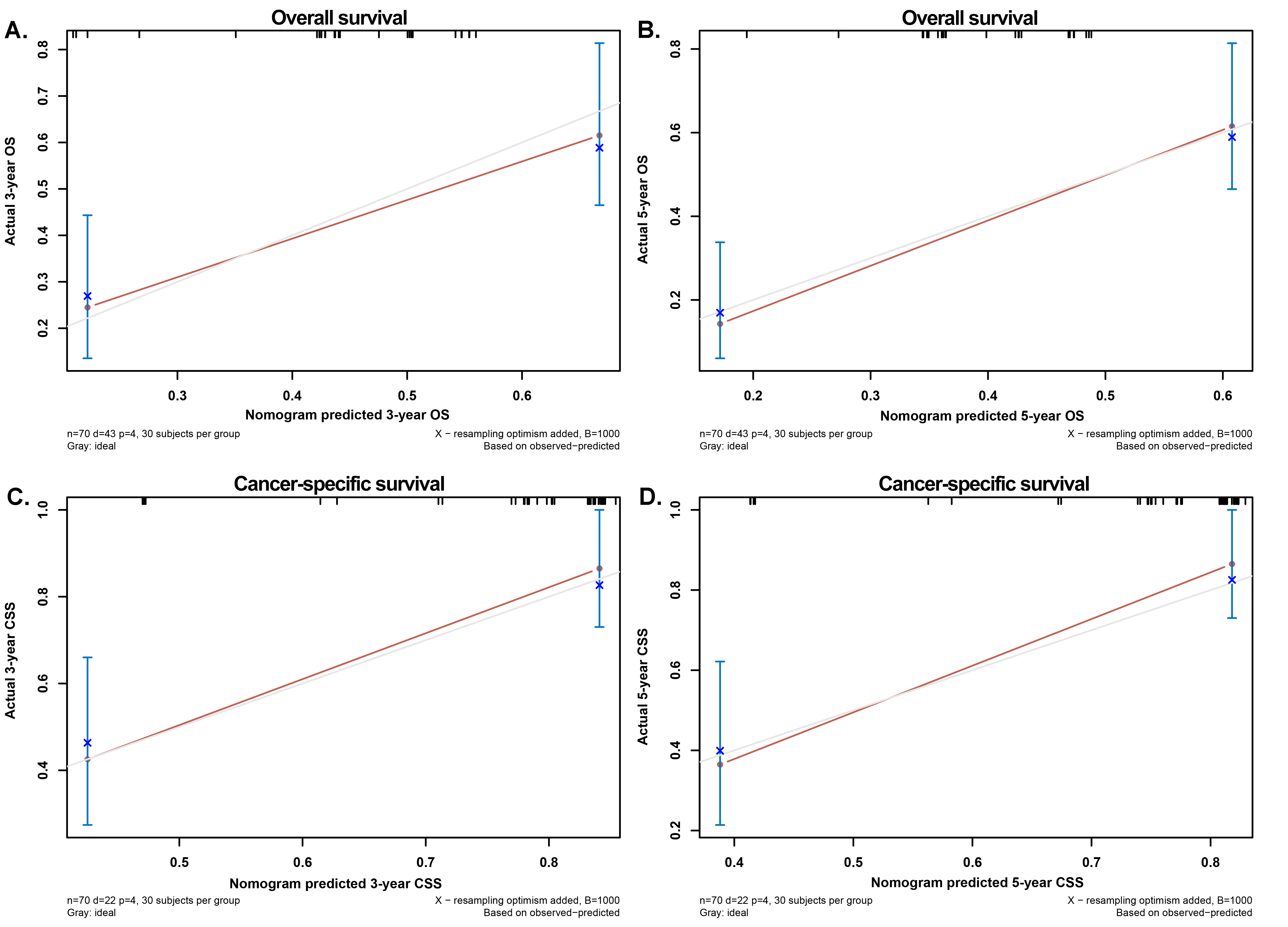

Supplement: Supplementary Figure 1 — Calibration curves for verifying the consistency between predicted 3-,5- OS and CSS and actual 3-,5- OS and CSS in the validation cohort. 3- OS (A) and 5- OS (B) calibration curves; 3- CSS (C) and 5- CSS (D) calibration curves. [file Image_1.JPEG]
